# Case report: dyslipidaemia—dramatic increase in haemoglobin A1c following statin initiation

**DOI:** 10.1093/ehjcr/ytae436

**Published:** 2024-08-27

**Authors:** Crystal B Chen, Marwan Badri, Erik Kelly

**Affiliations:** Lankenau Heart Institute, Lankenau Medical Center, 100 E Lancaster Avenue, 356 Medical Office Building East, Wynnewood, PA 19096, USA; Lankenau Heart Institute, Lankenau Medical Center, 100 E Lancaster Avenue, 356 Medical Office Building East, Wynnewood, PA 19096, USA; Lankenau Heart Institute, Lankenau Medical Center, 100 E Lancaster Avenue, 356 Medical Office Building East, Wynnewood, PA 19096, USA

**Keywords:** Case report, Statin-associated side effects, Diabetes mellitus, Hyperlipidaemia, Coronary artery disease

## Abstract

**Background:**

Statin therapy is associated with an increased risk of hyperglycaemia and new-onset diabetes mellitus. The absolute increase in glycosylated haemoglobin (HgbA1c, a measure of average glucose level over the past three months) is typically small; dramatic and clinically relevant increases are rare.

**Case summary:**

A 52-year-old man of South Indian descent with a history of hyperlipidaemia was started on rosuvastatin 40 mg daily for primary prevention of atherosclerotic cardiovascular disease. He did not have a history of diabetes mellitus. He developed polyuria and weight loss within weeks of starting statin therapy. Laboratory assessment was notable for HgA1c of 12.4% and LDL cholesterol of 84 mg/dL. Rosuvastatin was discontinued. He was not started on antidiabetic therapy as there was suspicion that statin therapy was the culprit for his HgbA1c rise. He soon had symptom resolution, and follow-up HgA1c 3 months later was 5.5%. Two years later, patient presented to the hospital with an acute coronary syndrome. He was discharged on rosuvastatin 40 mg daily and developed polyuria 1 week later. Rosuvastatin was discontinued, and atorvastatin 40 mg daily was initiated. Antidiabetic therapy was not started. He had resolution of his symptoms; follow-up HgA1c was below the diabetes threshold.

**Discussion:**

Statins are associated with a small increased risk of developing diabetes mellitus. The beneficial effects of statins on cardiovascular events typically outweigh any increased risk conferred by hyperglycaemia. While high-intensity statin therapy is routinely used as initial therapy for secondary prevention, we have no documentation explaining the choice of high-intensity statin for primary prevention in this case.

**Learning points**

- Statins are associated with a typically modest increase in serum glucose, and patients should be informed of this potential effect prior to drug initiation.
- In most patients, the benefits of statin therapy outweigh the risks of increased serum glucose.
- Dramatic elevations in HgbA1c following statin initiation, as described in this report, are rare.

## Introduction

Statins effectively reduce LDL cholesterol (LDL-C) and are widely used in the primary and the secondary prevention of atherosclerotic cardiovascular disease. Randomized controlled trials and observational studies have demonstrated an association between statin use and the development of new-onset diabetes mellitus in patients without pre-existing diabetes, with an incidence of approximately 9–25% compared with placebo.^1,2^ Patients with pre-existing diabetes have also been shown to experience a modest increase in glycosylated haemoglobin (HgA1c, a measure of average glucose level over the past three months) of 0.3% when treated with statins.^3^ We present a case of dramatic HgbA1c rise after the initiation of statin therapy and subsequent management decisions.

## Summary figure

**Table:** 

Time	Event	HgA1c (%)	LDL-C (mg/dL)
Day 1	Patient started on rosuvastatin 40 mg daily	∼5	170–180
Month 3	Patient reported polyuria within weeks of starting rosuvastatin	12.4	84
Month 6	Rosuvastatin was stopped. He was not started on pharmacotherapy for the treatment of diabetes mellitus. All symptoms resolved. Follow-up HgA1c obtained	5.5	
Month 21	Patient followed up for routine laboratory studies	5.6	140
Month 28	Patient presented to the hospital with an ST elevation myocardial infarction. Labs were obtained. He was discharged on rosuvastatin 40 mg daily. One week later, he developed polyuria. Rosuvastatin was stopped. Atorvastatin 40 mg daily was started	5.9	196
Month 30	Polyuria resolved and the patient denied new cardiopulmonary symptoms	5.6	54

## Case presentation

A 52-year-old male of South Indian descent with a history of hyperlipidaemia (off-treatment LDL-C 170–180 mg/dL) and baseline HgbA1c ∼5%, acid reflux, and vitamin D deficiency was started on rosuvastatin 40 mg daily by his primary care provider for primary prevention of atherosclerotic cardiovascular disease. We do not have documentation as to why high-intensity statin therapy was chosen. Patient had a family history of premature cardiovascular disease. His brother had a stroke in his 20s. His mother had a myocardial infarction in her 60s. The patient did not have metabolic syndrome or diabetes. His baseline body mass index was 24 kg/m^2^. At the time, his other medications included pantoprazole 40 mg twice daily and vitamin D3 50 mcg once daily. He noted polyuria and weight loss within weeks of starting rosuvastatin. He reported no other side effects. Three months after the initiation of rosuvastatin, his HgbA1c was 12.4% and LDL was 84 mg/dL. He was not started on treatment for diabetes mellitus, but rosuvastatin was discontinued. Other causes for hyperglycaemia such as stress hyperglycaemia, infection, and pancreatic disease were considered, though the patient had no clinical signs or symptoms to suggest these diagnoses. His symptoms resolved shortly after statin discontinuation. He had little healthcare contact over the next several months. A repeat HgbA1c at least 3 months later was 5.5%, and no other further testing was pursued.

Approximately 2 years later, the patient presented to the hospital with an ST elevation myocardial infarction. He underwent coronary angiography and percutaneous coronary intervention. On hospital presentation, his physical exam was notable for corneal arcus. He did not have any xanthomas. His body mass index was 25 mg/m^2^. His total cholesterol (TC) was 260 mg/dL, LDL-C 196 mg/dL, HDL cholesterol (HDL-C) 52 mg/dL, triglycerides (TG) 61 mg/dL, HgbA1c 5.9%, and creatinine 0.9 mg/dL. He was discharged on rosuvastatin 40 mg daily for secondary prevention of atherosclerotic cardiovascular disease based on the 2018 American Heart Association/American College of Cardiology Guideline on the Management of Blood Cholesterol.^4^ His treatment team was unaware of his prior experience with rosuvastatin. At a follow-up clinic visit 1 week later, his vital signs were within normal limits. His cardiopulmonary exam was unremarkable. The patient reported recurrent polyuria. Rosuvastatin was discontinued. He was started on atorvastatin 40 mg daily. Soon thereafter, his polyuria resolved, and he had no new symptoms. Repeat laboratory assessment 2 months later showed TC 107 mg/dL, LDL-C 54 mg/dL, HDL-C 38 mg/dL, TG 73 mg/dL, and HgbA1c 5.6%. The patient received counselling on lifestyle and dietary modifications for his pre-diabetes and dyslipidaemia. For his history of severe hypercholesterolaemia, he underwent familial hypercholesterolaemia genetic testing, which revealed no pathogenic sequence variants. He continues to demonstrate clinical stability without recurrent cardiovascular events.

## Discussion

The current European Society of Cardiology Guidelines on Dyslipidaemias discuss the modest increase in HgbA1c observed with statin therapy and the potential for new-onset diabetes mellitus.^5^ Statin use is associated with a 9–25% increased incidence of new-onset diabetes mellitus.^1,2^ However, the absolute change in HgbA1c is typically small.^3,6,7^ Indeed, some trials have not reported diabetes as a significant side effect of statin therapy.^8,9^ The JUPITER trial was the first randomized controlled trial to report a statin-associated increase of diabetes incidence. In this trial, patients without diabetes risk factors treated with rosuvastatin had an average HgA1c of 5.8% compared with 5.7% in the placebo group. In those with diabetes risk factors, the average HgA1c was 6.0% in the rosuvastatin group compared with 5.9% in the placebo group.^1^

The precise mechanism by which statins increase glucose levels is not fully understood, though several mechanisms have been proposed. One proposed mechanism is that statin-mediated inhibition of 3-hydroxy-3-methylglutaryl coenzyme A (HMG-CoA reductase) also decreases ubiquinone and isoprenoid synthesis. Ubiquinone depletion has been shown to impair insulin production. Isoprenoids are involved in glucose transporter type 4 expression in adipocytes, and depletion results in decreased glucose uptake.^9,10^ Statin-induced changes in immune response and upregulation of nitric oxide may also have negative effects on beta cell function.^10,11^ More potent statins at higher doses have been associated with new-onset diabetes. A recent meta-analysis of 19 trials found that low- or moderate-intensity statins resulted in a 10% proportional increase in new-onset diabetes vs. 36% in patients receiving high-intensity statins.^12^ Another meta-analysis found that rosuvastatin 20 mg daily compared with placebo was associated with a 25% increase in diabetes, while pravastatin 40 mg daily compared with placebo was associated with a 7% increase in diabetes.^13^ A similar association has been found in patients with established diabetes. In a pairwise meta-analysis, Cui *et al.*^6^ found a significant increase in HgA1c when statins as a new class were compared with placebo in diabetic patients (standardized mean difference 0.11). There was also an increase in HgA1c with high-intensity atorvastatin compared with placebo (standardized mean difference 0.63). It has also been postulated that lipophilic statins (atorvastatin, simvastatin, and pitavastatin) may be more likely than hydrophilic statins (rosuvastatin and pravastatin) to affect pancreatic beta cells.^11,14^

Clinicians must weigh the risk of new-onset diabetes, often with a modest increase in HgA1c, against the cardiovascular benefits afforded by statin therapy. Statins have been shown to reduce cardiovascular events in multiple patient subgroups, including those with diabetes mellitus.^11^ Therefore, the beneficial effects of statins on cardiovascular events and mortality typically outweigh an increased risk conferred by the development of serum glucose elevation or diabetes mellitus. Indeed, current guidelines do not support discontinuing statins if diabetes does develop.^8,15^

To our knowledge, there have been no published reports of dramatic increases in HgA1c after the initiation of statin therapy. While guidelines suggest continuing statin therapy in the setting of new-onset diabetes mellitus, given the dramatic HgA1c increase in this case, the decision was made to transition to an alternative high-intensity statin. Due to the patient’s high cardiovascular risk, complete discontinuation of statin therapy was not considered in accordance with guideline recommendations. Fortunately, the patient’s glycaemic control improved on atorvastatin. Interestingly, it was atorvastatin, a lipophilic statin at a high intensity that resulted in improved glycaemic control, suggesting that there is interpatient variability in the extent of glycaemic dysregulation with various statins. He has been maintained on the same statin dose with prolonged clinical stability.

## Conclusion

Statins confer a small increased risk in developing diabetes mellitus, though the average increase in HgA1c is typically modest. We present a case of a dramatic rise in HgbA1c after initiation of rosuvastatin 40 mg daily that improved with atorvastatin 40 mg daily. The beneficial effects of statins on cardiovascular events typically outweigh any increased risk associated with hyperglycaemia. Clinicians and patients should understand this potential side effect of statin therapy and how it should be managed.

## Data Availability

The data underlying this article are available in the article and in its online supplementary material. Additional information can be shared on reasonable request to the corresponding author.

## Lead author biography

Dr Crystal Chen is a graduate of Sidney Kimmel Medical School in Philadelphia. She completed her cardiovascular disease fellowship training at Lankenau Medical Center in Philadelphia. She is currently a non-invasive cardiologist at Virtua Health System in New Jersey.

**Consent:** The authors confirm that written consent for submission and publication of this case report including image(s) and associated text has been obtained from the patient in line with the COPE guidelines.

**Funding:** None declared.
